# The morphology of midcingulate cortex predicts frontal-midline theta neurofeedback success

**DOI:** 10.3389/fnhum.2013.00453

**Published:** 2013-08-09

**Authors:** Stefanie Enriquez-Geppert, René J. Huster, Robert Scharfenort, Zacharais N. Mokom, Johannes Vosskuhl, Christian Figge, Jörg Zimmermann, Christoph S. Herrmann

**Affiliations:** ^1^Department of Psychology, European Medical School, Carl von Ossietzky UniversityOldenburg, Germany; ^2^Karl-Jaspers Clinic, European Medical School, Carl von Ossietzky UniversityOldenburg, Germany; ^3^Research Center Neurosensory Science, Carl von Ossietzky UniversityOldenburg, Germany

**Keywords:** neurofeedback success, brain structure, fm-theta enhancement, midcingulate cortex, cingulate bundle

## Abstract

Humans differ in their ability to learn how to control their own brain activity by neurofeedback. However, neural mechanisms underlying these inter-individual differences, which may determine training success and associated cognitive enhancement, are not well-understood. Here, it is asked whether neurofeedback success of frontal-midline (fm) theta, an oscillation related to higher cognitive functions, could be predicted by the morphology of brain structures known to be critically involved in fm-theta generation. Nineteen young, right-handed participants underwent magnetic resonance imaging of T1-weighted brain images, and took part in an individualized, eight-session neurofeedback training in order to learn how to enhance activity in their fm-theta frequency band. Initial training success, measured at the second training session, was correlated with the final outcome measure. We found that the inferior, superior, and middle frontal cortices were not associated with training success. However, volume of the midcingulate cortex as well as volume and concentration of the underlying white matter structures act as predictor variables for the general responsiveness to training. These findings suggest a neuroanatomical foundation for the ability to learn to control one's own brain activity.

## Introduction

One of the most promising neuroscientific approaches for the enhancement of cognition and task performance, and eventually even for the treatment of psychiatric mental disorders, is neurofeedback (e.g., Enriquez-Geppert et al., 2013a). Neurofeedback is a form of brain computer interfaces (BCI); whereby neural parameters are fed-back to a human subject for up- or down regulation of one's own brain activity (e.g., Hinterberger et al., 2003; Bauerfeind et al., 2011; Scherer et al., 2013). Neurofeedback has been shown to be effective in the therapy of, among other conditions, attention deficit hyperactivity disorder (ADHD; e.g., Birbaumer et al., 2009) leading to a significant reduction in symptom severity even lasting for more than 2 years (Gani et al., 2008). Improved cognition and neurophysiological functioning due to neurofeedback training has also been demonstrated in healthy subjects (e.g., Egner and Gruzelier, 2001). Neurofeedback is based on trial-to-trial modulations of the ongoing neural activity, for instance on the modification of cortical oscillations (e.g., Enriquez-Geppert et al., 2013b). Participants play an active role while utilizing the real-time feedback based on the online-analysis of the brain activity in order to learn how to influence their own brain functions. This feedback loop hence adheres to operant conditioning principles. Finally, neurofeedback constitutes an inexpensive and non-invasive intervention tool.

Recent years have seen significant advances in technology and the investigation of cortical oscillations which have also impacted the advancement of neurofeedback approaches. For example, oscillations have gained much interest as a manifestation of neural mechanisms enabling brain communication and cognition (e.g., Herrmann et al., 2004). It has been shown that oscillations index sensory and cognitive processes, as for example seen with stimulus-induced amplitude changes (e.g., Basar et al., 2001). Other examples are augmentations of power in theta and alpha frequency bands with increasing task demands (e.g., Klimesch, 1999; Mitchell et al., 2008; Cohen and Cavanagh, 2011). Thus, the identification and usage of a particular oscillation, corresponding to a specific cognitive process that is to be modified, is of crucial importance for any neuroscientific intervention. For example, Keizer et al. (2010) revealed different behavioral effects of two neurofeedback protocols to increase either local gamma band or local beta band activity associated with a long-term memory task. The former modulation led to improved recollection, the latter to improved familiarity. Further emphasizing causal relationships between brain oscillations and cognition, it was shown that the manipulation of slow potential oscillations enhances memory retrieval after learning (Marshall et al., 2006), or that detection thresholds of auditory stimuli depend on the phase of an entrained 10 Hz oscillation during which stimuli are presented (Neuling et al., 2012).

While traditionally the number of neurofeedback training sessions is relatively high in clinical studies (up to 30 or 40 sessions), the utilization of individualized neurofeedback in healthy participants has been shown to succeed with substantially less sessions. Individualized neurofeedback refers to the individual determination of a specific frequency band for each participant. Frequency bands have been shown to vary considerably between subjects as a function of age, neurological diseases, and brain volume (Klimesch, 1999). Thus, individually determined frequency peaks might differ substantially between subjects, and may well be located at or beyond the border of another frequency band. Hence, using roughly fixed frequency bands irrespective of a single subject's brain state might not be optimal for neurofeedback training success. With this in mind, Zoefel et al. (2010) demonstrated the relevance of individualized alpha band neurofeedback training for the performance in mental rotation tasks with only five training sessions.

Despite the general success of neurofeedback trainings, studies often report a substantial amount of so-called non-responders: participants who do not show any modulation of their brain activity in response to a neurofeedback protocol (e.g., Hanslmayr et al., 2005; Doehnert et al., 2008; Zoefel et al., 2010; Enriquez-Geppert et al., 2013b). In 1995, Lubar and colleagues (Lubar et al., 1995) reported that 37% of their sample did not show modulated EEG activity after 40 sessions of neurofeedback training. Furthermore, in a continuous performance task on attention, retested after the training, these non-responders showed less improvement of performance than responders. Among others, Fuchs et al. (2003) have reported a similar subgroup that was not able to control their EEG activity after a comparably long training duration of 36 sessions.

Other studies have even reported a complete lack of behavioral effects with non-responders. For instance, in a study by Hanslmayr et al. (2005), neurofeedback success was strongly correlated with the enhancement of cognitive performance in a mental rotation task in healthy subjects. However, non-responders did not show any performance improvements. Similarly, Drechsler et al. (2007) reported neurofeedback training of slow cortical potentials in children with ADHD. Again, a subgroup of participants neither learnt to control their EEG during the course of neurofeedback nor did they show a decrease of clinical symptoms. Only the responders' training success was correlated with the regulation of disrupted behavior concerning the clinical symptoms of inhibition and impulsivity.

However, far too little attention has been paid to assess or even to predict and to explain non-responsiveness to neurofeedback (exceptions are Kotchoubey et al., 1999; Neumann and Birbaumer, 2003). One of the few studies investigating neurofeedback success focusing on sensori-motor rhythms (SMR; 12–15 Hz) was published by Weber et al. (2011). Here, early training success was positively correlated with the ability to regulate one's own brain activity at the end of training. Weber et al. (2011) further presented a classification scheme to determine responders vs. non-responders early in training. While this work is highly relevant for practical reasons, namely the possibility to early assign participants to appropriate interventions, still the mechanisms underlying these differences were not clarified. Addressing differences in individual strategies for EEG modulations, Nan et al. (2012) analyzed the association of mental strategies with training success in an upper alpha band neurofeedback training study, and reported strategies related to positive thinking to be the most successful ones. As suggested by Gruzelier et al. (1999), based on results of their study with schizophrenic patients who were less efficient in controlling their slow potential inter-hemispheric asymmetry, a lapse of concentration may as well play a role for decreased training success. In healthy participants, however, motivation and commitment to training may be more important for training success. To investigate this issue, Enriquez-Geppert et al. (2013b) assessed motivation and commitment by subjects' self-reports. However, a lack of motivation or commitment was not observed with non-responders.

A neglected issue with regard to the understanding of neurofeedback success concerns possible structure-function associations. That is, structural determinants of oscillations that are critical to neurofeedback success have rarely been focused on up to now. Recently, Halder et al. (2013) reported that deep white matter structures were correlated with BCI-performance.

With regard to the domain of behavioral training, more studies on brain function and learning success have been conducted. For instance, correlations between learning outcome and brain structure have been shown for language learning (e.g., Loui et al., 2011; Wong et al., 2011) and video game skill acquisition (e.g., Raz et al., 2000; Basak et al., 2011). In a grammar learning study, integrity of white matter tracts near the left Broca's area was correlated with training success (Flöel et al., 2009). In a video game skill training study, the volume of the dorsal striatum was correlated with enhanced gaming performance at the end of training (Erickson et al., 2010).

An oscillatory candidate for neurofeedback targeting the enhancement of high-order cognitive processes, such as executive functions, is frontal-midline (fm)-theta. Executive functions are known to enable the planning, controlling and monitoring of complex, goal-directed behavior and thoughts (Seiferth et al., 2007) and are associated with various behavioral and neurocognitive impairments when disrupted (e.g., Goldberg and Seidmann, 1991). When cognitive processing is enhanced, fm-theta shows an increased amplitude (Mitchell et al., 2008). Fm-theta enhancement has furthermore been related to event-related brain potentials; the so-called fm-negativities that are commonly observed in different tasks involving interference in information processing [e.g., the N200, the event-related negativity (ERN); Falkenstein et al., 2000; Nigbur et al., 2011]. Thus, fm-theta has been proposed as a universal mechanism for action monitoring with the midcingulate cortex (MCC; often referred to as dorsal anterior cingulate cortex) acting as hub for the integration of information (Cavanagh et al., 2012). However, a significant degree of inter-individual variability has been found concerning the peak frequency of fm-theta, while intra-individually the fm-theta frequency was found to be constant across time (Näpflin et al., 2008).

Regarding fm-theta neurofeedback, inter-individual differences concerning the ability to enhance their individual fm-theta amplitude have been shown, and non-responders have been identified as well (Enriquez-Geppert et al., 2013b). A main question therefore is, whether differences in the morphology of putative fm-theta generators are related to the ability to up-regulate brain activity utilizing neurofeedback. Related work showed that increased amplitudes of the N200 in the context of conflict monitoring tasks were found to be related to grossy-morphometric characteristics of the cingulate cortex, such as the occurrence of a second cingulate gyrus (Huster et al., 2007, 2009a,b, 2012). However, it has also been suggested that the MCC serves as a hub for information flow, using fm-theta to functionally interact with other cortical and subcortical areas (e.g., Cohen, 2011; Cavanagh et al., 2012). Thus, on the one hand, learning to increase fm-theta amplitudes might depend on the ability to recruit or synchronize the activity of midcingulate neurons, whose numbers are associated with regional volume and gross-morphology; on the other hand, white matter characteristics, such as increased bundle volumes, axonal density, or myelination, may ease the interregional synchronization of fm-theta oscillations (Cohen, 2011).

The aim of this paper is to determine potential relationships between fm-theta neurofeedback success and brain structures known to be critically involved in the generation of fm-theta. We built upon an individualized, eight-session fm-theta neurofeedback training. First, it is assessed whether early responsiveness to fm-theta neurofeedback is related to final training outcome as was already shown for SMR (Weber et al., 2011) and slow cortical potentials (Neumann and Birbaumer, 2003). Then, the association of structural characteristics of the MCC and fronto-cortical areas with fm-theta training success is examined. More specifically, it was investigated whether gray and white matter volume or concentration is associated with training success.

## Materials and methods

### Participants

Nineteen healthy participants took part in the experiment (11 women; mean age: 24 years, standard deviation: 2.7 years). We used the data of 14 subjects of our previous study (Enriquez-Geppert et al., 2013b) and acquired new data from another five subjects in order to have sufficient number of subjects for the current analysis. They were all right-handed as indicated by the Edinburgh Handedness Inventory (Oldfield, 1971), with a normal or corrected to normal vision. All gave written consent to the protocol approved by the ethic committee of the University of Oldenburg, and received a monetary reward of 8 € per h. The study was conducted in accordance with the Declaration of Helsinki.

### Study procedure

First, participants were invited for two separate sessions that were scheduled in rapid succession. During the first session, structural MR-images were taken at a local hospital (Pius Hospital, Oldenburg, Germany) according to the protocol specified below. On a second appointment, which took place at the Department of Psychology of the University of Oldenburg (Germany), a cognitive test-battery of executive function tasks (comprising the stop-signal, Stroop, n-back, task-switching task) with concurrent EEG recordings was performed in order to identify the individual's dominant fm-theta frequency. The individual fm-theta frequency was subsequently used for an individualized neurofeedback. These measurements were always done on the first working day of a week. The actual neurofeedback training started the following day (Tuesdays). The individualized fm-theta neurofeedback training consisted of eight sessions in total, spread over the course of 2 weeks, ending the Thursday of the second week.

### MRI scanning protocol

The acquisition of the individual anatomical scans was obtained on a 3 Tesla MRI scanner (Siemens Magnetom Verio) equipped with the standard bird cage head coil at the Pius Hospital of Oldenburg, Germany. Participants' heads were stabilized by using foam cushions to minimize movement-related artifacts. Generalized Autocalibrating Partially Parallel Acquisition was used to obtain 176 contiguous T1-weighted (*TR* = 1900 ms, *TE* = 2.52 ms) 1 mm thick slices in the sagittal plane with a 256 × 256 matrix size and a field of view of 250 × 250 mm^2^, resulting in an in-plane resolution of 0.98 × 0.98 mm^2^.

### Processing of MR-images

First, the subjects' images were individually co-registered and normalized to MNI reference space. Images were then segmented using the iterative unified segmentation approach. In addition to concentration values, modulated images were computed to allow for voxel-wise volumetric comparisons. In short, modulated images are computed by scaling the concentration images based on parameters of previous normalization steps such that the total amount of regional gray matter is preserved. Concentration and modulated volume images were computed for both gray and white matter. Data were smoothed with a Gaussian kernel of 8 mm FWHM. Details on the exact procedures can be found in Ashburner and Friston (2009) and the SPM8 manual available at www.fil.ion.ucl.ac.uk/spm.

### Region of interest analyses

After processing of the images for each subject, mean values for a selected number of cortical areas were computed by masking images with regions of interest (ROI) derived from gray matter and white matter atlases specified below. Gray matter regions were selected according to their relevance for being potential generators of fm-theta. According to Mitchell et al. (2008), at least two sources of fm-theta are to be differentiated. The MCC has been implicated to be the main generator of fm-theta, especially in context of demanding cognitive tasks relying on cognitive control. On the other hand, regions of superior, middle, and inferior frontal cortices have been suggested to either generate or to rely on fm-theta as transfer signal. Hence, for each hemisphere the MCC, superior and middle frontal cortices, as well as pars triangularis, orbitalis, and opercularis of the inferior frontal cortex (IFC) were extracted using the templates provided by the software package Automated Anatomical Labeling (AAL; Tzourio-Mazoyer et al., 2002). Whereas one might argue that the power of fm-theta activity relies on gray matter characteristics such as the number of synchronized neurons, the individually dominant frequency, for example, rather might rely on features of underlying white matter tracts, reflected in the cingulate bundle (e.g., Valdés-Hernández et al., 2010; Nunez, 2011; Zaehle and Herrmann, 2011). The cingulate bundle is located below the MCC in its rostro-caudal course and is supposed to consist of associative fibers originating and terminating in cingulate regions, but also connecting to the prefrontal cortex (Schmahmann et al., 2007). Similarly, bilateral superior longitudinal fascicle II (SLF) were extracted, which represent an interhemispheric bi-directional tract connecting the prefrontal with the parietal cortex (e.g., Makris et al., 2005). Lastly, bilateral white matter structures of the anterior and superior corona radiate were extracted that connect the prefrontal cortex with the thalamus and deep brain structures (Mori et al., 2005; Schmahmann and Pandya, 2006). White matter ROIs were extracted from the JHU white-matter tractography atlas (Hua et al., 2008).

### EEG recordings, cognitive test battery, and neurofeedback training

All EEG recordings were performed in an electrically shielded and sound attenuated room using the Brain Vision Recorder software in combination with BrainAmp EEG amplifiers (Brain Products GmbH, Gilching, Germany). Electrode impedances were kept below 5 kΩ with nose as an online reference.

Before neurofeedback started, the individual fm-theta frequency of each subject was determined based on the estimation of the cognitive test battery of executive function tasks. (1) A visual number-letter task was applied for task-switching. Numbers had to be classified into even or odd, letter into vowels or consonants via a right or left hand button press. Depending on the background color, participants had to switch between number and letter processing (switch condition) or to continue (no switch condition). (2) An alternating three-back-, zero back task was used for memory updating, containing letters as stimuli. During the three-back task, participants were instructed to identify those letters, which were presented exactly three trials before (updating condition). During zero-back task, participants had to indicate those letters presented at the beginning (no updating condition). (3) A visual stop-signal task was applied for response inhibition. In the majority of trials, participants reacted with left and right button presses (go condition) according to left and right arrows, however, whenever as stop-signal followed, announced by a color change of the arrows, subjects had to abort their initiated response (stop condition). (4) For conflict monitoring the Stroop task was utilized. Color names were presented and participants had to respond according to the ink color of the color names via left hand or right hand button press. The ink colors either corresponded to the presented color names (congruent condition) or not (incongruent condition). During task processing, EEG was recorded from 32 electrodes while subjects performed the cognitive test battery. Preprocessing of the concurrently recorded EEG data was accomplished by means of EEGLAB (freely available from http://www.sccn.ucsd.edu/eeglab). EEG recordings were filtered with low-pass (80 Hz) and high-pass filters (0.5 Hz), and down-sampled to 250 Hz. Eye artifacts were corrected by an infomax independent component analysis (ICA; Bell and Sejnowski, 1995; Makeig et al., 2004), and incorrect responses were discarded. For each the inhibition condition of the stop-signal task, the conflict condition of the Stroop task, the updating condition of the three-back task, and the switching condition of the task-switching task, individual fm-theta frequency peaks were identified in the range of 4–8 Hz of the event-related spectral perturbation (ERSP). These task-specific fm-theta frequencies were averaged and used for the individualized neurofeedback training.

For the neurofeedback, EEG activity was recorded at seven electrodes (Fz, FC1, FCz, FC2, Cz, Fp1, and Fp2) with an online nose reference and read out in real-time. Data was processed by in-house software programmed in Matlab 7.14 (the MathWorks, Natrick, USA) by performing Fast Fourier-Transforms (FFT) with a hamming window every 200 ms on a sliding 2 s data window. During training sessions, ocular artifacts were detected at Fp1 and Fp2. Before each training session, an EOG calibration was conducted to support the detection and the rejection of eye blinks. During this EOG calibration, time frames containing eye artifacts were identified based on an individually adapted amplitude threshold. Artifact-specific peak amplitudes were then identified and extracted from an FFT. Based on this individual frequency peak, an individualized eye artifact-related frequency band was calculated and used during the following measurements in order to reject every trial (2 s data window) containing artifacts. Feedback was given in form of a colored square presented on a computer desktop. To increase individual fm-theta activity, participants were instructed to color the square red as long and saturated as possible by applying mental strategies (e.g., mental operations, emotions, imaginations etc.).

At the beginning of each session, the above mentioned EOG calibration was performed. The neurofeedback training sessions themselves consisted of 6 training blocks (block 1–6) each lasting five min with short breaks in between. Before and after these training blocks, further 5 min blocks were recorded; these were the so-called start and end baseline measurements during which no feedback was given. During these measurements the color of the square slowly altered between red, gray, and blue. Participants were instructed to count the red gradients, but not to use any mental strategy and to rest with eyes open. The start baseline measurement of each particular training session was used as reference for the feedback in the training blocks. During training blocks, the square turned gray when there was no difference from baseline or eye artifacts were detected. The feedback color ranged from a highly saturated red over gray to a highly saturated blue with a total of 40 color steps and depended on the actual fm-theta activity. The feedback color was updated every 250 ms and changed to red when the fm-theta power was enhanced, and changed to blue when the power was decreased relative to fm-theta start baseline power. Ninety-five percent of the power range was covered by the feedback saturation scale. Values above 97.5% or below 2.5% were indicated by maximal red or blue saturation, respectively.

### Dependent variables and statistical analyses

In accordance with Dempster and Vernon (2009) neurofeedback success was assessed using two different learning indices, each quantifying a potentially distinct aspect of training success. Concretely, the first learning index (L1) captures dynamical changes within neurofeedback sessions and therefore reflects the immediate responsiveness to neurofeedback. For the quantification of L1, training power was extracted for each training block (1–6) and baseline measurement (start, end baseline) and averaged across all training sessions as fm-theta changes relative to the power observed during the first start baseline measurements. In contrast, the second learning index (L2) focuses on the maintenance of such enhancements from session to session and captures changes over the whole course of training. Therefore, L2 reveals rather lasting training effects. To achieve L2, effects over the course of training were compared to brain activity at the beginning of the training. Frequency changes of each training session were quantified as changes relative to the first training session. Both, L1 and L2 were calculated with respect to changes in μV and show differences between participants belonging to the actual NF group and the pseudo-NF control group (Enriquez-Geppert et al., 2013b).

### Training effects on fm-theta frequency

To test if there were training effects in L1 within sessions compared to the baseline measurements, a repeated-measures ANOVA with the factor *block* (start baseline, block 1, block 2, block 3, block 4, block 5, block 6, end baseline) was calculated for the changes in μV. Concerning the potential increase of fm-theta over the course of training, L2 was analyzed by means of a repeated-measures ANOVA with the factor *session* (one to eight) for changes calculated in μV.

### Correlation of early and final training success

To investigate if early training effects are related to the final training success, a Pearson product-moment correlation was computed for fm-theta changes of the second and the eighth session (after testing for normal distribution by the Kolmogorov-Smirnov Test). The correlation was tested by means of a one-tailed *t*-test for significance using SPSS Statistics 20 (SPSS, Chicago, USA). Learning index L2 was applied.

### Multiple regression analyses for predicting training success by brain structure

To investigate whether training success was associated with volumes and concentration measures of gray and white matter ROIs, four different regression analyses were conducted using a stepwise method in order to test which set of variables significantly predicts training success. For each calculation of the learning index (L1, L2) two regression analyses were set up. One regression analyses used gray matter ROIs as predictor variables, the other white matter ROIs (corresponding volume and concentration measurements were entered into one model; see Table 1). The regression models were tested for significance using SPSS Statistics 20 (SPSS, Chicago, USA). Standardized beta values (β) and corresponding significance values are reported as well.

**Table 1:** **Overview of multiple regression analyses**.

**Model**	**Criterion**	**Significance**	**β-values**	**Significant predictor variable**
1	L1 in μV	*p* < 0.01	0.63	vol., r. MCC
2	L1 in μV	*P* < 0.01; *p* < 0.001	0.681;0.681	conc., r. cingulate bundle and vol. cingulate bundle
3	L2 in μV	n.s.	/	/
4	L2 in μV	n.s.	/	/

*Four regression analyses were conducted on the learning indices L1 and L2 as criterion variables. These criterion variables were predicted on the basis of gray and white matter ROIs (volume and concentration measurements). For each analysis the significance, the standardized β values, and the significant predictor variables are listed. Abbreviations: vol., volume; conc., concentration; r., right; l., left*.

## Results

### Fm-theta increases within sessions and accumulates over the course of training

**Figure 1:**
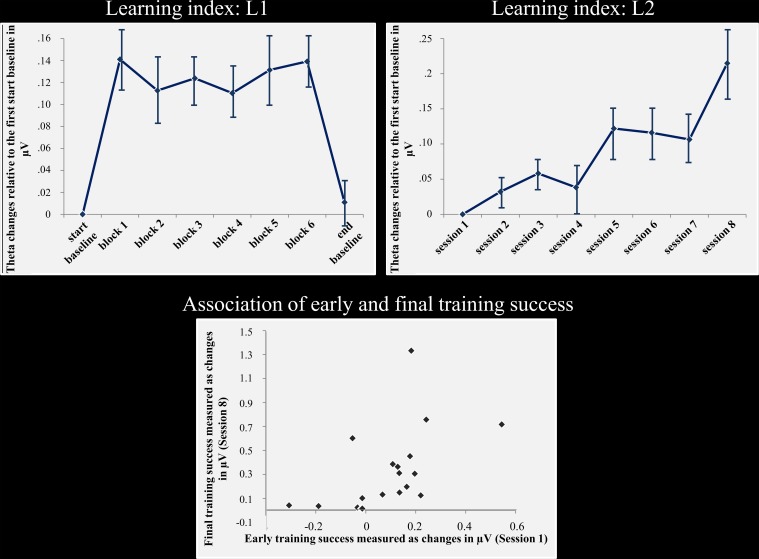
**Neurofeedback success and association of early and final training success**. **Top:** the left column shows the fm-theta increase as reflected by the learning L1. This index reflects amplitude changes calculated across all training sessions for the start baseline measurement, blocks 1–6, and the end baseline measurement. During feedback, fm-theta is strongly enhanced compared to the start and end baselines. The right column depicts the fm-theta changes during the course of training, beginning with sessions 1–8. During the course of training, fm-theta enhancements are accumulating with each training session. Means and standard errors of means are depicted. **Bottom:** Training induced fm-theta enhancements are depicted for the second and the eights session for each participant. The figure illustrates the strong relationship of early and final training success. The stronger the fm-theta increase is at the beginning of the training, the better the final training outcome.

Figure 1 depicts fm-theta changes within sessions across all training days (L1), as well as the alterations over the course of the training (L2). Indeed, a significant amplitude increase can be observed within the neurofeedback sessions relative to the baseline measurements. Similarly, amplitude increases from session to session are highlighted as well. Statistical analyses did confirm these observations. The main effect *block* was significant for the repeated-measures ANOVA testing changes in L1 [*F*_7, 126_ = 18.882, *p* < 0.001]. The main effect *session* of the repeated-measures ANOVA testing for theta frequency power changes over the course of training (L2), was as well-significant [*F*_(7, 126)_ = 6.315, *p* < 0.001].

### Early neurofeedback success correlates with final training success

As expected, there was a positive and highly significant correlation between training success in the second and eighth session (*r* = 607; *p* < 0.01; see Figure 1) for L2. Thus, the final ability to control one's own brain activity after training was positively correlated to initial training success.

### Predictions of training success from brain structure

An overview of the results obtained by the multiple regression analyses is presented in Table 1. From these data it can be seen that no significant model was revealed when testing L2. However, strong evidence with regression models for dependent variables L1. Therefore, statistics assessing effects on L2 will not be reported.

#### Predicting L1 from gray matter characteristics: the right MCC predicts fm-theta increase within sessions

**Figure 2:**
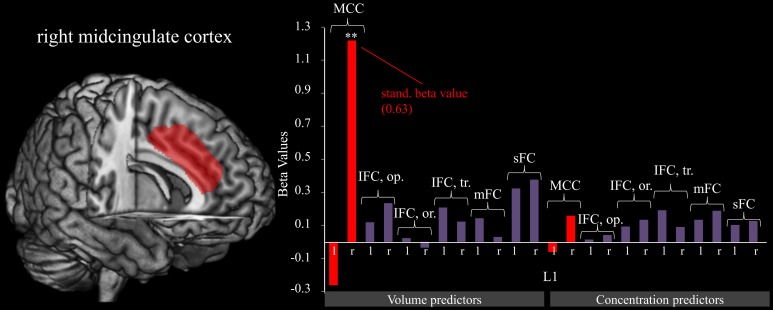
**Right midcingulate cortex predicts L1**. The graphic shows the relation of fm-theta increases during training sessions (L1). Predictor variables include gray matter volumes (left) and concentrations (right). The figure reveals that the volume of the MCC contributes strongly to training success as is shown by the significant (^**^p < 0.001) beta value, whereas all other brain structures show a negligible relationship. For significant predictors, standardized beta values are included as well.

A significant model emerged using the stepwise method [*F*_(1, 17)_ = 11.186, *p* < 0.01, adjusted *R*^2^ = 0.361] in which the gray matter volume of the right MCC (see Figure 2) significantly predicted the gain of dynamical changes (β = 630, *p* < 0.01). No other gray matter ROIs were predictive.

#### Predicting L1 from white matter characteristics: the cingulate bundle predicts fm-theta increase within sessions

**Figure 3:**
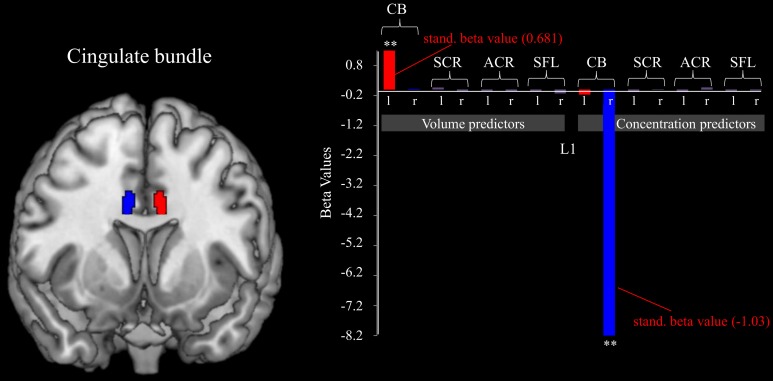
**Cingulate bundle morphology predicts L1**. The figure shows the relation between the volumes and concentrations of white matter belonging to different brain tracts and the fm-theta increases during training sessions (indexed by L1). The volume of the left, and the concentration of the right cingulate bundle act as significant (^**^p < 0.001) predict variables of the ability to increase fm-theta during training sessions. No further white matter structures influence training success. Standardized beta values are included for the significant predictor variables.

Similar effects were also found for the white matter below the midcingulate cortex. Utilizing the stepwise method for white matter ROIs as possible predictor variables for the gain of dynamical changes resulted in a significant model [*F*_(2, 16)_ = 24.651, *p* < 0.001, adjusted *R*^2^ = 724]. Significant predictors were the concentration within the right cingulate bundle (β = −1.03, *p* < 0.001) and the volume of the left cingulate bundle (β = 0.681, *p* < 0.001; see Figure 3).

## Discussion

Neurofeedback has been suggested as a powerful neuroscientific approach to modulate cognition, task performance, and clinical symptoms. Prior studies on neurofeedback revealed substantial inter-individual differences in training success, even reporting non-responders, i.e., participants who are not at all able to modulate their brain activity (e.g., Hanslmayr et al., 2005; Doehnert et al., 2008). One goal of this study was to assess whether early training outcome within an eight-session neurofeedback training study would be correlated with the finally achieved fm-theta enhancement. The results show that already in the second training session the amount of fm-theta enhancement indicates the finally achieved fm-theta augmentation observed at the end of training. Importantly, these data suggest that early training responsiveness serves as predictor for the ultimate training success. These findings are in agreement with observations of Weber et al. (2011), who focused on SMR neurofeedback.

However, from a neuroscientific perspective it is of substantial interest to describe neuronal aspects underlying neurofeedback success. Up until now, nothing has been reported in the literature regarding potential factors contributing to neurofeedback training success. Therefore, this study mainly set out to assess the relationship between preexisting structural differences and the modulation of fm-theta by neurofeedback. It was reasoned that preexisting inter-individual differences in the morphology of brain structures involved in the generation of fm-theta may as well be associated with training success. Hence, volume and concentration measures of gray and white matter ROIs in midcingulate and prefrontal regions were used as predictors for neurofeedback training success. Two types of learning indices were chosen. While the first index focuses on the ability to enhance fm-theta within a training session, the second describes the maintenance of enhancements from one training session to the next and thus stresses gradual changes over the whole course of training. Larger volumes of the right MCC, as well as higher white matter concentration of the right and larger volumes of the left cingulate bundle were associated with stronger fm-theta enhancement during training sessions as reflected by the first learning index (L1). However, the ROIs did not predict lasting training success as reflected by L2. Thus, known brain structures acting as fm-theta generators seem to play only a minor role with respect to L2. Possibly other brain regions involved in learning and memory of operant conditioning may be of more importance regarding the maintenance of training enhancements. Thus, different aspects of neurofeedback training may be associated with morphological differences in diverse functionally specialized brain structures.

With respect to the neurofeedback learning indices, it is important to consider that these were shown to differentiate between the actual neurofeedback group and an active control, the so-called pseudo neurofeedback group. In a previous study we found that the pseudo group showed no enhancement of L1 at all, and significantly weaker non-specific effects in L2 (Enriquez-Geppert et al., 2013a). Importantly, this pseudo protocol led to similar experiences of pseudo subjects concerning commitment, motivation, and task difficulty (Enriquez-Geppert et al., 2013a). Together these findings suggest that the L1-related changes do not simply reflect increased task difficulty due to the use of mental strategies to enhance fm-theta during training.

The observed associations between gray and white matter structure and the enhancement of fm-theta during the actual training sessions were in line with hypotheses based on potential generators of fm-theta. Several studies using intra-cerebral recordings in animals and humans concluded that fm-theta has its main source in the MCC (Wang et al., 2005; Tsujimoto et al., 2009; Womelsdorf et al., 2010). Also, source localization of scalp-recorded fm-theta in healthy subjects consistently indicates the MCC as major generator (Iramina et al., 1996; Gevins et al., 1997; Asada et al., 1999; Ishii et al., 1999; Onton et al., 2005; Sauseng et al., 2007).

Thus, the association of inter-individual differences of MCC volumes, cingulate bundle volumes, and concentrations with fm-theta responsiveness to training was expected and confirmed. Larger volumes seem to facilitate the possibility to modulate fm-theta oscillations. The reverse suggestion might be that small midcingulate volumes imply reduced success during feedback sessions. Differences in MCC morphology have already been reported in relation to neurofunctioning and performance. Earlier work already indicated that the MCC shows a high degree of structural variability in terms of gray and white matter volumes, local gyrification or regional microstructure (e.g., Yücel et al., 2001; Fornito et al., 2004; Huster et al., 2007, 2009a,b). In general, gyrification has been shown to have a functional significance (Welker, 1990), and to be affected by the underlying cytoarchitecture (e.g., Watson et al., 1993), as well as the neural connectivity (Caviness et al., 1989). Vogt et al. (1995) showed that the variation of the gyral pattern is associated with differences in the size and distribution of cytoarchitectonically defined regions within the MCC. When present, the PCG comprises area 32′. It has been suggested that the presence of the PCS may be a consequence of stronger internal connectivity within this area, whereas the absence may be related to stronger external connectivity (Welker, 1990). The degree of midcingulate fissurization was demonstrated to relate to differences in behavior, as well as to neuropsychological functioning in executive tasks (Fornito et al., 2004; Huster et al., 2009a,b, 2012). Some of these studies also showed that subjects with a leftward midcingulate folding asymmetry exhibited increased electrophysiological reactivity in tasks calling for cognitive control.

Structure-function associations have also been revealed for micro-structural properties of the white matter utilizing diffusion tensor imaging (DTI) and event related potentials (ERPs). Findings indicate that increased amplitudes of electrophysiological activity coincide with augmented efficiency of brain communication, as for example seen with the ERN, an ERP associated with an erroneous response. The ERN is suggested to reflect theta oscillations and to be generated in the MCC (e.g.,Trujillo and Allen, 2007; Cavanagh et al., 2012). Westlye et al. (2009) investigated inter-individual differences of ERN amplitudes and fractional anisotropy (FA), a measure providing information about white matter microstructure, such as axon caliber, fiber density, or myelination. Interestingly, associations of higher ERN amplitudes and increased FA values were observed in the left cingulate bundle (Westlye et al., 2009). Likewise, Cohen (2011) found higher theta power to be associated with stronger tract connectivity between regions underlying EFs such as the MCC, the striatum, and the ventrolateral frontal cortex. Similarly, measures indexing the transmission of signals across the corpus callosum, as the so-called inter-hemispheric transfer time (IHTT), have been found to be associated to micro-structural properties of the corpus callosum as well. The IHTT is computed by analyzing latency-differences of sensory evoked potentials between contralateral and ipsilateral electrodes when presenting stimuli to only one hemisphere. This way, Westerhausen et al. (2006) showed a negative correlation between the IHTT and the mean diffusivity in the posterior third of the corpus callosum. Likewise, Withford et al. (2011) predicted the IHTT from FA and mean diffusivity.

When analyzing the association of training effects and brain structure, at least two issues arise. In case of the current study, the ability to enhance one's own fm-theta power might be based on larger number of neurons in the MCC. However, from a microscopic perspective ample possible cellular and molecular factors exist that contribute to variations in volume or structural concentrations. Among them are variations in the amount of dendritic or axonal arborization, synaptic connections, vascularization, as well as differences in the number of neurons that all can also lead to differences at the functional level. When finding associations of training success and brain structure, further issues arise. For example, such effects may well be caused by common genetic or environmental enrichment effects on both brain structure and training success. The engagement in physical activities (e.g., Kramer et al., 1999) and the engagement in cognitively challenging events (Hertzog et al., 2009) may serve as examples for environmental effects, both of which have shown to also affect neuroplasticity.

In conclusion, inter-individual differences concerning the ability to change one's own brain states are in focus of current neurofeedback research. By analyzing the associations of training success and pre-existing inter-individual differences in midcingulate morphology, we elucidated putative neuroanatomical foundations for the ability to learn to control one's brain system ultimately causing fm-theta generation.

### Conflict of interest statement

The authors declare that the research was conducted in the absence of any commercial or financial relationships that could be construed as a potential conflict of interest.
